# A Lab-on-a-Chip for the Extraction and Analysis of Single Molecules of DNA from Biological Media

**DOI:** 10.3390/nano16120732

**Published:** 2026-06-12

**Authors:** Franziska M. Esmek, Louise von Lacroix, Lucjan Grzegorzewski, Irene Fernandez-Cuesta

**Affiliations:** 1 Institute of Nanostructure and Solid State Physics, Universität Hamburg, HARBOR Bldg 610, Luruper Chaussee 149, 22761 Hamburg, Germany; louise.lacroix@uni-hamburg.de (L.v.L.); lucjan.grzegorzewski@uni-hamburg.de (L.G.); 2 Hamburg Centre for Ultrafast Imaging, 22761 Hamburg, Germany

**Keywords:** microfluidic, nanofluidic, lab-on-a-chip, DNA extraction, DNA analysis, optical readout, single molecule analysis

## Abstract

DNA extraction is a critical prerequisite for reliable downstream analyses such as Polymerase Chain Reaction (PCR), sequencing, and genotyping. Conventional methods often require labor-intensive protocols, large sample volumes, or costly automation. Microfluidic approaches offer an alternative by reducing reagent consumption and enabling faster, more integrated workflows. Here, we present a passive lab-on-a-chip device that performs DNA extraction from complex biological media and enables subsequent on-chip single-molecule analysis. The chip integrates a magnetophoresis-based solid-phase extraction module with a fluorescence detection section capable of quantifying DNA molecules in microchannels and visualizing stretched molecules in nanochannels. The multi-level micro/nanofluidic architecture is fabricated in polymer using a single-step nanoimprinting process with a total manufacturing time of two minutes per chip, enabling scalable production. As a proof of concept, the device extracted DNA from samples spiked into buffer or plasma. On-chip transfer efficiency of DNA–bead complexes to the elution buffer reached 86%, and quantitative analysis of the recovered liquid showed an overall extraction efficiency of 40% (including DNA recovery off-chip), with intact 48 kbp DNA confirmed in both micro- and nanochannel measurements. This platform offers a promising foundation for point-of-care and point-of-interest applications, where integrated DNA extraction and analysis can reduce sample loss and support streamlined, automated workflows.

## 1. Introduction

DNA analysis is key in molecular biology, genomics, forensics, and several other areas of life sciences [1,2,3,4] where DNA extraction from the sample is a fundamental and indispensable first step. For example, for clinical diagnosis, tumor DNA or pathogen genetic material has to be extracted from patients’ samples, like tissues or body fluids. For environmental monitoring, DNA can be extracted from freshwater samples to enable early monitoring of harmful cyanobacterial blooms before toxin concentrations reach hazardous levels. In forensic contexts, DNA is extracted from trace biological evidence such as blood, saliva, hair follicles, or skin cells collected at crime scenes. In many of such applications, DNA extraction is initiated by cell lysis, which disrupts cellular and nuclear membranes to release nucleic acids into solution. However, lysis alone is insufficient, as the resulting lysate contains a complex mixture of proteins, lipids, polysaccharides, and enzymatic inhibitors that can interfere with downstream molecular assays, so the DNA has to be extracted into a “clean” liquid buffer. In other applications, like liquid biopsy, cell-free DNA can be directly extracted from the liquid sample without the lysing step.

Efficient extraction of DNA molecules from the complex biological media like the cell lysate, blood or other body fluids, fresh water or mud guarantees high-purity samples for downstream analyses such as PCR, sequencing or genotyping [5,6,7,8]. This ensures consistent and reproducible results, as removing other biological molecules, impurities, and contaminants reduces variability between experiments and increases the reliability and sensitivity of the molecular assays [9,10].

There are already many commercial methods for extracting DNA from liquid samples, which are regularly used in laboratories [11]. However, they involve bulky, expensive robotic equipment or labor-intensive protocols with multiple steps and significant manual work. In addition, they often require relatively large sample volumes (typically 100–500 µL for commercial spin-column kits) due to their limited sensitivity. And due to sample pipetting and manipulation, material losses are still a problem [12]. As a result, low-abundance genetic material, trace pathogens, or subtle genomic alterations are still difficult to detect [13,14].

Solid-phase extraction (SPE) is a standard method used for extracting and purifying DNA from various liquid sources [15]. For this, a solid-phase material, usually functional beads or a porous membrane, is used to capture DNA molecules from the liquid selectively. This method usually consists of three steps: first, the solid material (e.g., beads) is mixed with the complex biological media, so that the DNA molecules bind to the beads’ surface. Second, a series of washing steps is performed to remove salts, proteins, and other impurities from the sample. And third, after all impurities have been washed away, the DNA molecules are eluted from the solid phase material into a (clean) buffer [16].

Microfluidic chips are powerful platforms for manipulating minute amounts of liquid. They offer numerous advantages over conventional methods, such as miniaturization, low losses, faster processing times, and improved automation, and all require significantly lower sample volumes [17,18,19,20,21,22,23]. Interestingly, solid-phase materials can be integrated into microfluidic chips by adapting the protocol steps for on-chip implementation, thereby enabling rapid and selective DNA extraction [24,25]. These devices have the potential to be portable and to allow DNA extraction and purification in situ, in the field, from minute amounts of liquid and with minimal losses. In addition, this will improve sample quality, since purified DNA reduces degradation and loss before it arrives at the research laboratory for further analysis [17,26].

The integration of DNA purification with subsequent analytical techniques, such as PCR or sequencing, also on-chip, would push DNA analysis several steps further [27,28,29]. This would allow fully automated workflows on miniaturized chips and in portable systems, with few handling steps, resulting in minimal DNA loss and increased overall sample quality, enabling the analysis of scarce material [30].

Conventional DNA analysis workflows are typically two-step: extraction is performed first—in a vial or on a spin column—and the purified DNA is then transferred to a separate analytical instrument such as a spectrophotometer (NanoDrop), fluorometer (Qubit), gel electrophoresis system, or automated capillary electrophoresis platform (TapeStation) for quantification or size analysis. Each transfer step introduces the risk of sample loss and contamination, and the instruments involved are not compatible with point-of-care or field-deployed settings. Moreover, bulk ensemble instruments such as NanoDrop and Qubit provide total DNA mass but no information on fragment length distribution; gel electrophoresis and TapeStation provide length information but require nanogram-to-microgram quantities per lane, cannot be integrated into a closed fluidic workflow, and give population-averaged results rather than single-molecule resolution. The platform presented here is designed to integrate this two-step workflow into a single passive device in which both, extraction and single-molecule characterization, occur sequentially without any sample transfer. This is directly relevant to point-of-care diagnostics, forensic trace DNA analysis, and liquid biopsy, where sample volumes are limited and every handling step represents an unacceptable risk of loss.

In this work, we have developed a lab-on-a-chip based on an integrated micro- and nanofluidic device for DNA extraction and subsequent analysis. The designed chip consists of two parts: the purification part and the analysis part. In the purification step, DNA molecules are extracted from the biofluid using SPE with magnetic beads. The subsequent analysis is performed using a laser system. Here, the DNA molecules are fluorescently labeled, stretched in nanochannels, and read out in real time by a focused laser [31]. The single-molecule nanochannel analysis methodology—laser-assisted detection of molecules (LADOM)—has been developed and validated by our group in prior work, including studies of DNA conformation in nanochannel confinement and the effect of the inlets on molecular flow [32,33,34] and the use of optical mapping and barcoding for applications [31,35]. The contribution of the present paper is to couple this established analytical capability with a new on-chip DNA extraction module, creating a fully integrated, passive lab-on-a-chip. For high scalability, the microfluidic chips are made exclusively of polymer and can be fabricated in a two-minute process by direct UV nanoimprinting [32,33,36]. We present a proof-of-concept lab-on-a-chip for the extraction and analysis of DNA spiked into buffer and human plasma.

## 2. Materials and Methods

### 2.1. Silicon Stamp Fabrication

**Figure 1:**
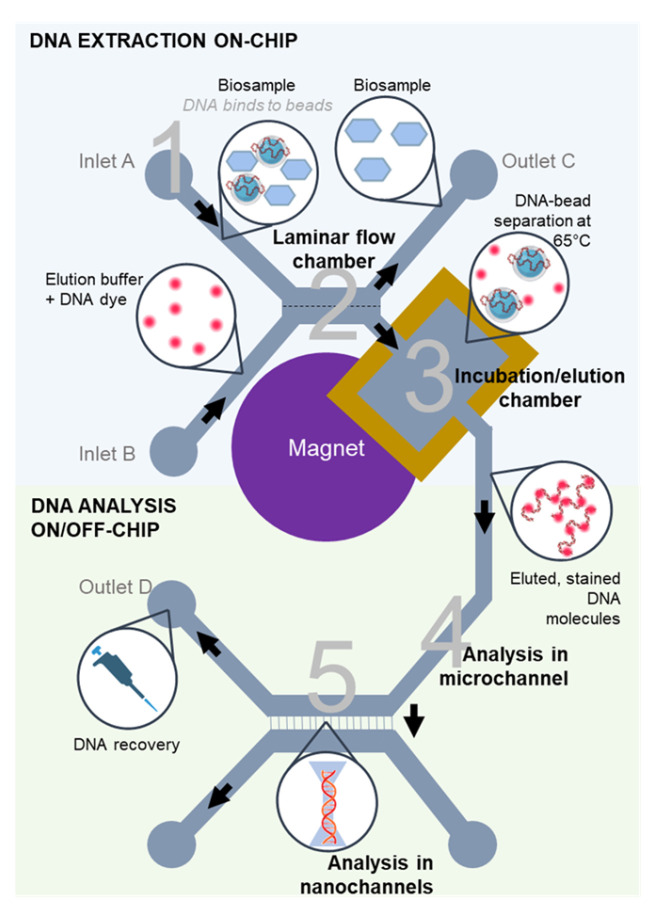
Schematic overview and concept of the micro- and nanofluidic chip for DNA extraction and analysis. (1) The mixture of liquid biosample with DNA molecules and magnetic beads and binding buffer is pipetted in inlet A. (2) In the laminar flow chamber, the microchannel from inlet A and the microchannel from inlet B with elution buffer and DNA dye run in parallel, in a laminar flow regime. A magnetic field pulls the DNA–bead complex from the upper stream and into the lower one. (3) In an incubation chamber, locally heated at 65 °C, the DNA is eluted from the beads and stained in situ by the dye in the elution buffer. Subsequently, the DNA can be analyzed on chip or collected in outlet D for off-chip analysis. (4) In the microchannels immediately after the elution chamber, a laser can be used to monitor DNA flow rate, enabling real-time tracking of the extraction process and estimating the concentration of DNA in the initial biosample. (5) In the nanochannels, it is possible to analyze the DNA with further detail, as the molecules are analyzed one by one as they flow through in a stretched conformation. This allows, for example, the fragment length distribution of the sample to be obtained.

**Figure 2:**
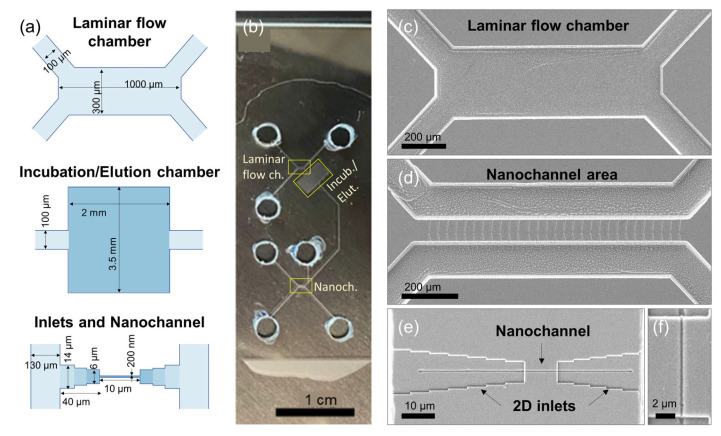
Images of the complete lab-on-a-chip device and details of its different parts. (**a**) Sketch of the main functional components of the lab-on-a-chip device, and their corresponding dimensions. (**b**) Photograph of an OrmoStamp device, all transparent and single-use, with the various sections visible and marked. The device is 1.5 cm × 3.5 cm. (**c**) SEM image of the laminar flow chamber in the silicon stamp, located within the extraction section of the chip, characterized by a width of 300 µm, a length of 1 mm, and a depth of 30 µm. (**d**) SEM image of the analysis section in a silicon stamp, which features microchannels, nanochannels and 2D inlets designed to facilitate a smooth transition between them. (**e**) A close-up view of a 2D inlet with staggered width and depth, and a nanochannel (**f**).

The silicon stamp was fabricated using a combination of photolithography and electron-beam lithography, first patterning the nanostructures (nanochannels) and then the microstructures (2D inlets and microchannels). The design of the chip can be seen in Figure 1, and images of the structures in Figure 2.

A silicon wafer (100) was spin-coated with polymethyl methacrylate (PMMA) (AR-671.06, from Allresists GmbH, Strausberg, Germany) at 4000 rpm for 1 min, then baked at 150 °C for 3 min. The nanochannels were then written by electron-beam lithography using a Voyager (Raith GmbH, Dortmund, Germany) electron-beam writer at 50 kV with a dosage of 250 µC/cm^2^. Following exposure, the resist was developed using the developer 600.55 (also from Allresists) for 1 min and stopped by rinsing into isopropyl alcohol (IPA). The nanostructures were etched into the wafer using an inductively coupled plasma reactive ion etching (ICP-RIE) process (SI500, SENTECH Instruments GmbH, Berlin, Germany) with the parameters listed in Table 1. There, SF_6_ chemically etches silicon via fluorine radicals, whereas C_4_F_8_ forms a fluorocarbon polymer layer that passivates the surfaces and promotes anisotropic etching with vertical sidewalls. Oxygen is added to control the fluorocarbon passivation by reducing excessive polymer deposition, thereby stabilizing the etch profile. To overcome the entropic barrier between the micro- and the nanostructures, we integrate gradient inlets connecting the microchannels and the nanochannels. These have gradually decreasing widths and/or depths, and help to increase the molecular flow throughput and to pre-stretch the molecules, decreasing clogging. In this work, we used 2D inlets, where we decreased the width from 14 µm to 6 µm, as opposed to 3D inlets used in other works, where both width and depth are varied simultaneously. These 2D inlets were fabricated in a similar manner to the nanostructures. The electron-beam lithography dosage was adjusted to 150–250 µC/cm^2^ to create steps of varying depths. The RIE parameters (Table 1) differ only in process time from those used for the nanochannels.

The microchannels were produced using photolithography and RIE using a hard mask. Copper (Cu) was sputtered onto the silicon wafer to a thickness of 100 nm. Subsequently, a layer of photoresist (S1813, Kayaku Advanced Materials, Inc., Westborough, MA, USA) was spin-coated onto the copper at 4000 rpm for 1 min and baked at 115 °C for 1 min. A photomask was aligned to the previously created nanostructures using a mask aligner (Karl Süss MBJ4, SUSS MicroTec SE, Garching, Germany) and exposed for 5 s. The structure was then developed in MF319 for 1 min and rinsed with water. The copper layer beneath the structure was selectively etched with an iron nitrate solution. The microchannels were then etched by RIE (Table 1). The depths of all structures were determined using a profilometer (DEKTAK, Veeco Instruments Inc., Tucson, AZ, USA) and from SEM images (Zeiss Crossbeam 550, Carl Zeiss Microscopy GmbH, Carl-Zeiss-Str. 22, Oberkochen, Germany).

### 2.2. Micro/Nanofluidic Device Fabrication

The fluidic devices were produced by direct UV nanoimprinting with the OrmoStamp polymer (micro resist technology GmbH, Berlin, Germany). The process starts with the fabrication of a negative polymer master stamp replicated from the silicon stamp. For this, a glass substrate was coated with OrmoPrime through spin-coating at 4000 rpm for 60 s, followed by baking at 150 °C for 5 min to create an adhesion coating. Liquid OrmoStamp was cast onto the silicon structure, and the glass plate with the OrmoPrime adhesion coating was placed on top. The assembly, comprising the silicon structure, liquid polymer, and the glass plate, was cured under UV light for 2 min. The glass plate, now bearing the cured OrmoStamp structure, was separated from the silicon wafer by placing them on a hot plate. The OrmoStamp surface was activated with UV-Ozone (Jelight Company, Inc., Irvine, CA, USA) for 2.5 min and subsequently coated with fluorosilane (1H,1H,2H,2H-Perfluoroctyltriethoxysilane) in a vacuum oven at 80 °C for 30 min. This negative master stamp was then utilized multiple times to imprint the single-use devices. For this, several drops of OrmoStamp were cast on the stamp. Then, a Plexiglas plate (74 mm × 26 mm) with 6 or 7 holes, designed for use as inlets and outlets for the liquid, was positioned on top of the liquid polymer and exposed to UV light for 2 min. Following exposure, the device was gently removed from the master stamp using a hot plate at 80 °C. The structure on the Plexiglas device was sealed by attaching a coverslip, also made from OrmoStamp material.

### 2.3. External Elements

Since device manufacturing is to be kept as simple as possible to allow easy fabrication of single-use fluidic chips; we used external elements (i.e., a permanent magnet and a heating element) placed outside the chip to generate magnetic fields or increase temperatures at specific positions on the chip. In this way, we can implement all the steps of the DNA extraction protocol within the chip.

To generate the magnetic field, an NdFeB permanent magnet was employed. The disk-shaped magnet had a diameter of 4 mm and a height of 2 mm. It possesses a magnetization grade of N45 and a maximum operating temperature of 80 °C. The device and the magnet were mounted on a 3D-printed holder, which allowed us to control the distance between the laminar flow chamber and the magnet.

**Figure A1:**
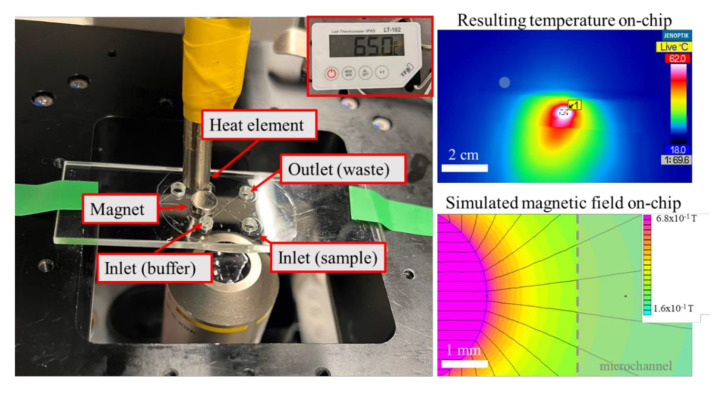
Setup of the external temperature and magnetic field elements. The sample is detected using an inverted fluorescence microscope, with external components including a heating cartridge for temperature control and a permanent magnet for generating the magnetic field. A thermal camera is used to measure the actual temperature, showing a heated area with a 5 mm diameter; the outline of the incubation/elution chamber (3.5 mm × 2 mm) is overlaid on the thermal image with a black dashed line, and named as region 1, confirming it is fully contained within the heated zone. The resulting magnetic field is simulated using FEMM 4.2, illustrating the forces as a function of distance from the permanent magnet.

The heating element is generated by a heating cartridge connected to a power source. A thermal camera was used to measure chip temperature and ensure that the heat was localized within the incubation chamber (see Appendix A, Figure A1). To generate a temperature of 65 °C on the chip, a voltage of 23 V and 65 mA must be set. The exact layout of the heating element and the temperature settings as a function of the voltage are provided in the Appendix A.1.

### 2.4. Detection Setup

The optical setup consists of an inverted fluorescence microscope (Nikon TiU, Nikon Corporation, Tokyo, Japan) into which two light sources (a focused laser and a widefield broadband lamp) are coupled. The He:Ne laser has a wavelength of 633 nm and a power of 20 mW. A Scientific Complementary Metal-Oxide-Semiconductor (sCMOS) camera (Sona, Andor Technology Ltd. (Oxford Instruments), Belfast, Northern Ireland, UK, or Kinetix, from Teledyne Photometrics, Tucson, AZ, USA) and a single-photon counter are coupled to the two outputs of the microscope. In front of the single-photon counter, a 100 μm-diameter pinhole is installed to physically confine the collected signal to 1 μm around the laser spot. We used a 10× air objective for imaging the overall flow, and a 100× oil-immersion objective with an NA of 1.45 for observing the DNA and for fluorescence measurements with the photodetector. In addition, two superimposed filter cube stages can either filter or let light pass through a specific wavelength range. For these experiments, a Cy5 filter cube (692/40 nm, from the Semrock Brightline series, IDEX Health & Science (Semrock), Rochester, NY, USA) was mainly used.

### 2.5. Quantification of Pulled-Down Beads

To determine how many DNA–bead complexes transition between streams in the laminar flow chamber, the sample is mounted on the microscope, and the area of the chamber imaged along time. The magnet is used for 30 min, and the percentage of beads pulled to the other flow stream was obtained from the recorded videos, analyzed using ImageJ (version 1.54g, W.S. Rasband, U.S. National Institutes of Health, Bethesda, MD, USA, https://imagej.net/ij/, accessed on 21 April 2026). A control experiment was conducted without a magnetic field. Over 30 min, the intensities of the chamber’s inputs and outputs were analyzed using ImageJ. Subsequently, the bead input was set to 100%, and the elution buffer input to 0% (background). The intensities of the output streams were then analyzed on this fixed scale. It should be noted that in all experiments, the establishment of laminar flow was first verified before we placed the magnet. As long as the chip remained intact, laminar flow was observed in 100% of cases, and the magnetic beads did not migrate into the other phase when the magnet was not used. In addition, the agglomeration rate was analyzed by measuring the area blocked by beads, which is then no longer accessible to the flow. This occurs when the magnetic force is too strong, pressing the beads against the wall and immobilizing them. In this situation, the magnetic force exceeds the hydrodynamic force, resulting in chamber blockage in the lower flow stream.

### 2.6. DNA Preparation and the Used Kit

For DNA purification on the developed chip, all reagents from the KIT “Dynabeads™ DNA DIRECT™ Universal Kit” from Thermo Fisher Scientific, Waltham, MA, USA were used.

Lambda DNA from bacteriophage lambda cl857 Sam 7 was used for all experiments, spiked in the different liquids. The DNA was purchased from Thermo Scientific. Lambda DNA (double-stranded, 48,502 base pairs) has a contour length of around 16 µm (when unlabeled), which can increase up to 20 to 22 μm when labelled with an intercalating dye at full intercalation (ratio of base pairs to dye of 3:1) [37,38]. The fluorescent dye used to stain the molecules was TOTO3 (Invitrogen™ (Thermo Fisher Scientific, Waltham, MA, USA) Dimeric Cyanine Nucleic Acid Stains), with absorbance and emission peaks at 642 and 660 nm, respectively, matching the laser used for excitation. TOTO3 has high affinity to double-stranded DNA, like most intercalating dyes, which could also be used in the platform. For tests requiring the detection of single-stranded DNA, it would be possible to use other dyes instead, like SYBR Gold or OliGreen. The buffer used to elute the DNA molecules from the magnetic beads is 0.25× TBE buffer. To aid the elution process, we heated the microfluidic chamber to 65 °C following the recommendations from the provider [39].

## 3. Results

### 3.1. Lab-on-a-Chip Concept

The chip, sketched in Figure 1, has two parts: one for DNA extraction and one for DNA analysis, all monolithically integrated within the same fluidic device.

#### 3.1.1. On-Chip Extraction of DNA

In the first section of the fluidic device, the DNA molecules are directly extracted from a biofluid by magnetophoresis. For this, the magnetic beads and a specific binding buffer are mixed with the target biofluid in a vial off-chip. As soon as the magnetic beads and the fluid are combined, the DNA binds to the beads’ surfaces via electrostatic interactions. Then, 10 µL of the fluid mixture in the vial is pipetted into inlet A (Figure 1). Simultaneously, a buffer with low ionic strength (0.25× TBE) containing an intercalating dye (TOTO3) specific to double-stranded DNA is added to inlet B (10 µL).

The two microchannels guide the liquids from the two inlets into a chamber (Step 2 in Figure 1), where the microchannels merge and run parallel before separating again. In this chamber, both fluids flow side by side in a laminar regime, unmixed. The laminar flow and the absence of mixing between the two liquids are essential to ensure that only the magnetic beads are separated from the non-purified, original solution and transferred into the clean elution buffer. At these length scales, we expect a negligible effect from diffusion, with a diffusion layer in the micron regime, which would not affect the buffer composition.

An external magnet is placed near the chamber at a specific position controlled by a 3D-printed holder. Thus, in the presence of the magnet, the beads get pulled down from the upper flow stream and into the lower stream with the clean buffer. Everything else contained in the biofluid (except the DNA–bead complex) is directed to outlet C and discarded.

After the laminar flow chamber, the bead–DNA complexes are guided to an incubation chamber (3, in Figure 1), where they get trapped by the field of the magnet. The chamber is heated to 65 °C by using an external heating element, thus promoting the detachment of the DNA from the beads. There, the DNA is eluted from the beads and labeled in situ by the intercalating dye present in the buffer. To control and calibrate the temperature at that specific position and monitor the extent of the heated area, the chip’s temperature was imaged using a thermal camera. The heat map images are available in the Appendix A (Figure A1).

After elution, the DNA molecules are guided into the analysis section along a microchannel (marked 4), where a laser is used to measure their concentration and length in real time at the single-molecule level. Alternatively, the extracted DNA can be recovered from outlet D and analyzed off-chip using standard commercial techniques.

#### 3.1.2. On-Chip Analysis of DNA

In the chip’s analysis section, DNA molecules can be analyzed in nanochannels using the previously developed laser-assisted detection of molecules (LADOM) method [31]. This technique, established in prior work by our group—covering optical mapping and barcoding [31,35], DNA conformation in nanochannel confinement [32], and passive flow characterization [34]—is used here as the analytical readout to verify the integrity and concentration of the extracted DNA molecules. For this purpose, several (29) nanochannels run perpendicular to the microchannel and are connected to them via 2D inlets, as shown in Figure 2d. These connect the wide microchannels to the much narrower nanochannels by providing a smooth transition in channel width. These inlets enhance molecular capture rates, pre-stretch the DNA molecules, and help to prevent clogging [32,40]. In part thanks to these structures, DNA molecules can spontaneously flow into the nanochannels, facilitating efficient single-molecule analysis [34]. This allows entirely passive device operation, since the liquid and the molecules flow and move without the need to apply pressure or an external field, which significantly simplifies overall operation. In the nanochannels (200 nm wide and 100 nm deep), the DNA molecules are stretched, and their genomic fingerprint and/or length can be read out using a laser. For this, a focused laser spot, 2 µm in diameter, is placed at the center of the nanochannel to excite the labeled molecules as they flow through, and a photodetector records the fluorescence in real time. In this way, we obtain step-shaped peaks in the time trace corresponding to single DNA molecules, with peak duration associated with the length of the specific molecule. In addition, as demonstrated previously, it is possible to create a genome-dependent barcode on the molecules using different labeling protocols, which would, in future work, allow the acquisition of a sequence-dependent fingerprint of the extracted molecules [31,35,41,42,43,44,45,46,47]. In addition, the DNA concentration can be estimated.

### 3.2. Device Fabrication and Operation

The fluidic chips are fabricated by direct UV nanoimprinting. For this, a silicon stamp is first fabricated and replicated (see Section 2). Once the stamps are ready, the actual process of fluidic device fabrication consists of dropcasting OrmoStamp and UV curing, and takes just two minutes, enabling high-throughput chip production. These fluidic devices have a footprint of 1.5 cm × 3.5 cm and are single-use and transparent, which is helpful for optical measurements.

**Figure A2:**
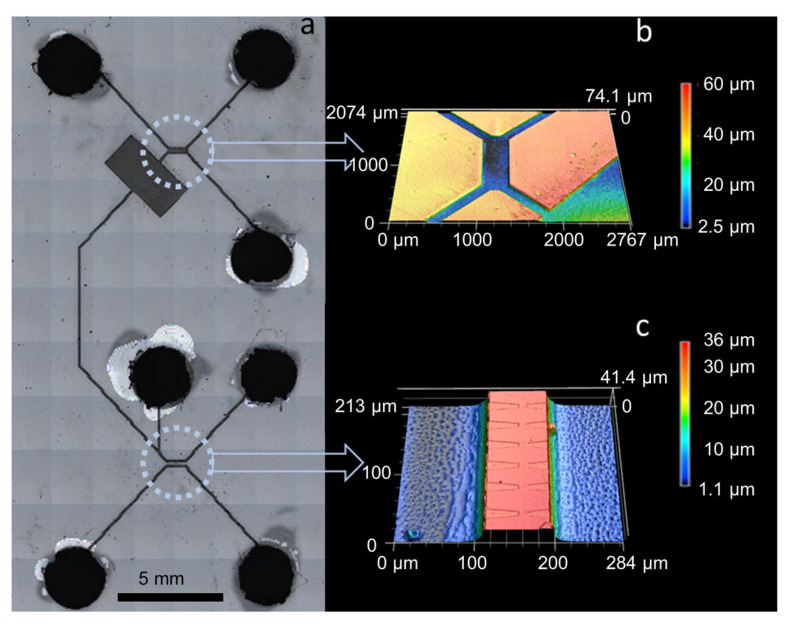
3D microscope images of the microfluidic device. (**a**) Overview of the microfluidic device, fabricated via a two-minute nanoimprint lithography process. (**b**) Close-up of the laminar flow chamber, showing a uniform depth of 30 µm, where magnetic beads are separated from the solution. (**c**) In the analysis region, nanochannels with a depth of 100 nm extend from the microchannel through 2D inlets.

The dimensions of the structures are sketched in Figure 2a: the microchannels have a depth of 30 µm and a width of 130 µm. The nanochannels are 200 nm × 100 nm in cross-section and 10 µm long. The funnel inlets have a cross-section of 6 µm × 200 nm at the tip and 14 µm × 800 nm at the broadest part. Figure 2 shows SEM images of the laminar flow chamber (Figure 2c) and the nanochannel area (Figure 2d–f) in a silicon stamp. One complete lab-on-a-chip micro- and nanofluidic device is shown in Figure 2b. More images of the device and the structures can be seen in Figure A2.

The device’s flow is purely passive, so we did not use tubing, fluidic holders, pumps, or electrophoresis during the experiments. In this configuration, where the liquid is dropped in the inlet with a micropipette and without using force, flow is driven by a combination of capillary pressure at the liquid–air interface in the channels, hydrostatic pressure from the liquid column at the inlet, and evaporation at the open outlet. The Reynolds number, Re, for flow in the microchannels was estimated from Equation (1):

(1) Re=ρvDh/μ

where ρ is the density of the fluid (≈1000 kg/m^3^ for our aqueous buffer), v is the flow speed (0.023 mm/s at the center of the microchannel, as measured by tracking the extracted DNA molecules flowing along a microchannel from the fluorescence videos), D_h_ is the hydraulic diameter (≈70 µm for the extraction chamber, 61 µm for the microchannels), and μ is the dynamic viscosity of the fluid (≈10^−3^ Pa·s for water at room temperature). This gives Re ≈ 0.0016 for the extraction chamber and Re ≈ 0.0014 for the microchannels, confirming fully laminar flow throughout the device, well below the turbulent transition threshold of Re ≈ 2300.

We used an external magnet and an external heating element (i.e., neither is an integral part of the devices). This has the advantage that fluidic device fabrication, preparation and operation remain as simple as possible, and each microfluidic device can be made in just 2 min and used once. A photo of the setup is shown in Figure A1. Thermal imaging was performed to ensure the elution chamber temperature was 65 °C, which is essential for efficient extraction and to prevent denaturation. This also helped us monitor that the heat remains confined to the chamber and does not propagate along the chip, preventing DNA elution from the beads before extraction.

### 3.3. Quantification of DNA Loss in Laminar Flow Chamber

To analyze how much DNA can be extracted and identify the critical steps where DNA molecules are lost along the process, different elements and steps were analyzed independently.

Initially, the DNA molecules bind to the surface of magnetic beads. This binding process is facilitated using the provided binding buffer, which, according to the provider, can bind up to 1 µg of DNA using 200 µL of beads from the stock. In our developed chip, we intentionally worked with significantly less DNA than the maximum capacity, utilizing 150 ng DNA in total. We assume that the losses associated with this initial step are negligible and that all the DNA in the sample binds to the beads.

**Figure 3:**
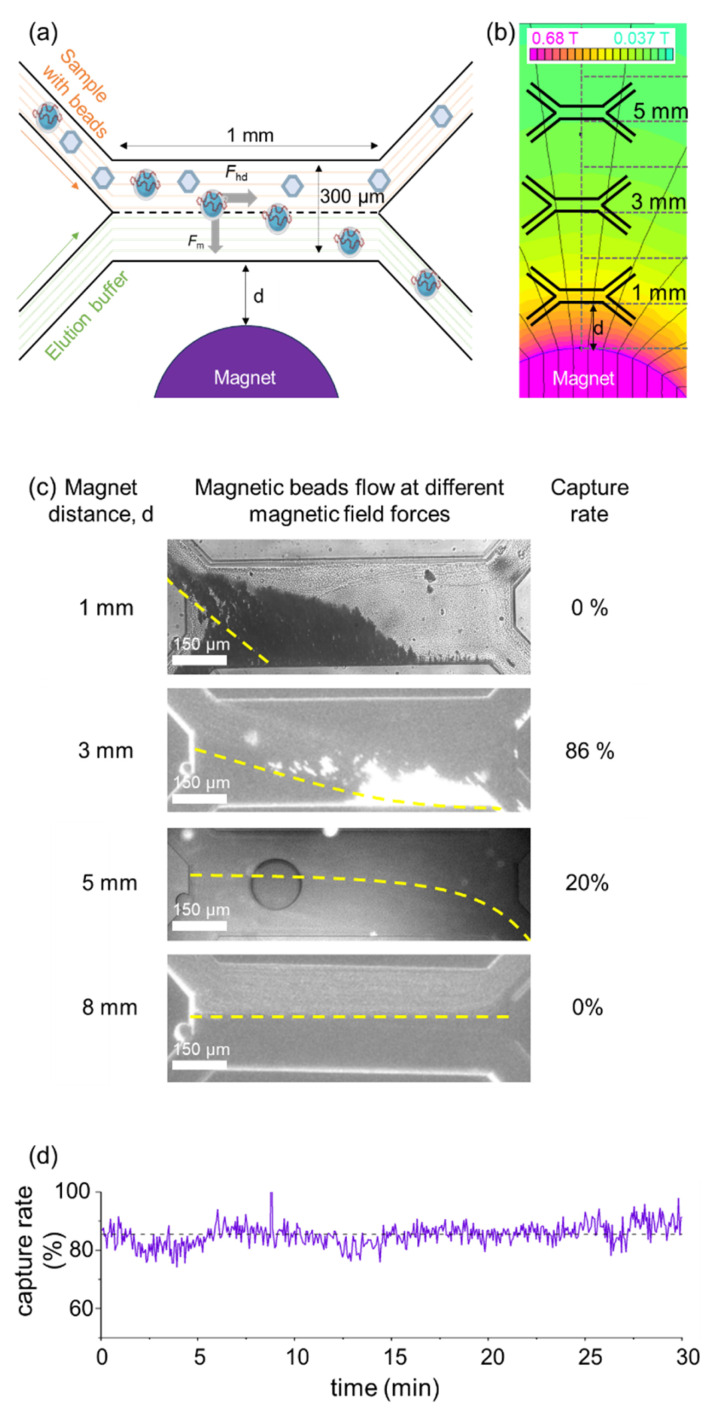
Influence of the applied magnetic field on the magnetic bead pull-down. (**a**) Sketch and concept of the magnetophoresis in the laminar flow chamber. (**b**) Simulated magnetic field acting at a 1, 3, and 5 mm separation on the flow of magnetic beads in the laminar flow chamber. (**c**) Images showing the effect of magnetic force on DNA–bead complexes in the chamber, observed by placing the magnet at different distances, along with their respective capture rates, calculated as the number of beads pulled down into the lower stream in a stable fashion. By positioning at 1 mm, 3 mm, 5 mm, and 8 mm, the beads are attracted to varying degrees by the permanent magnet. The optimal result is achieved at 3 mm, where most of the complexes are transferred without significant agglomeration. The images were obtained either with bright field in transmission (1 mm, 5 mm) or in epifluorescence (3 mm, 8 mm), since the magnetic beads show autofluorescence, which makes the tracing easier, especially when recorded in-flow. (**d**) Magnetic bead capture rate over time in the presence of the applied magnetic field over 30 min, resulting in an average capture rate of 85.6%.

Once the DNA molecules bind to the beads, the next step is to transfer the DNA–bead complexes from the binding buffer to the elution buffer in the chip using the magnet to extract the DNA from the biofluid. To estimate the magnetic force exerted by the magnet in the laminar flow chamber at various distances, we used FEMM (Finite Element Method Magnetics (FEMM), Version 4.2; Available online: http://www.femm.info, accessed on 21 April 2026) to simulate the magnetic field (Figure 3b).

To optimize the pull-down rate of beads into the elution buffer, we observed the chamber and the liquid and beads flow in the microscope while we placed the magnet at different distances (between 1 and 8 mm) from the center of the chamber (see Figure 3a,b). We measured how many beads were pulled down and how many remained in the upper stream for the different magnetic field strengths (see Section 2 and Appendix A.1).

Figure 3c shows images of the chamber for different magnet positions. At a distance of 1 mm (1050 nN), the magnetic beads are abruptly drawn into the lower channel. Although all magnetic beads were pulled into the lower stream, they agglomerated at the channel wall, diverting the elution buffer flow and causing a blockage or obstruction in the chamber. At a distance of 3 mm, a majority (86%) of the magnetic beads were pulled across and flowed with the elution buffer into the lower channel system. Despite the continued presence of a few beads agglomerating at the channel wall due to magnetic forces, the agglomeration rate was low enough not to affect the liquid flow. The microscope was used to monitor that bead clusters do not affect the flow streams. The stable capture rate throughout the 30 min experiment (Figure 3d) shows no downward trend that would be expected from progressive clogging, thus confirming the optimal flow. Increasing the distance to 5 mm or even 8 mm eliminated agglomeration at the channel walls, but only 20% (at 5 mm) or 0% (at 8 mm) of the beads were captured. The 3 mm distance yielded the best results and was thus set as the optimal, fixed distance in subsequent experiments.

Figure 3d displays the capture efficiency of the magnetic beads in the lower stream over 30 min. This shows that, with a constant magnetic field, the magnet’s capture efficiency remains constant over time. Under optimized conditions, on average, 85.6% of the DNA–bead complexes are drawn into the lower stream, which then flows into the incubation and elution chambers before the analysis section.

### 3.4. Quantification of Single DNA Molecules in the Microchannel to Monitor the Extraction Rate in Real Time

**Figure 4:**
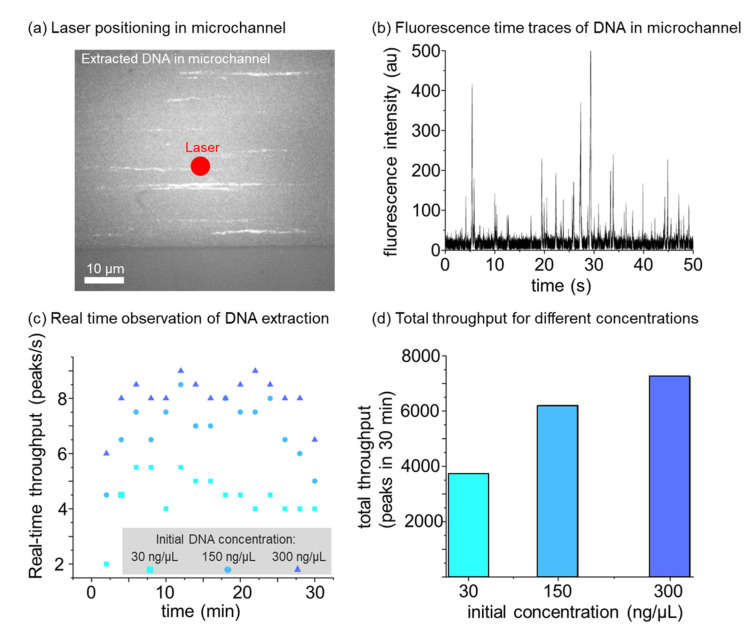
Analysis of DNA extraction in real time in the microchannel using a laser system. (**a**) A laser spot, 2 μm in diameter, was positioned within the microchannel through which the purified DNA flows. (**b**) A photon counter is used to record fluorescence intensity time traces, and each DNA molecule passing through the laser spot results in an event in the detector. (**c**) Peak throughput was obtained for samples with various initial DNA concentrations over 30 min, which can be used to monitor the extraction process in real time. (**d**) Total number of peaks counted across the three samples with different DNA concentrations, providing a semi-quantitative monitor of relative DNA throughput that depends on the initial DNA concentration in the liquid inserted into the device.

One of the first analyses we can perform with on-chip detection is to quantify and monitor the concentration of extracted DNA in the elution buffer in real time. With this, we can observe the extraction as it occurs within the chip, which helped us understand its functioning and dynamics. For this, a laser spot is positioned at a fixed location in the microchannel after the incubation/elution chamber (Figure 1, Section 4), as shown in Figure 4a. An example of a recorded intensity time trace is presented in Figure 4b, where the intensity peaks correspond to fluorescence outbursts from the DNA molecules as they pass through the laser spot. The fluorescence intensity is measured over time with a temporal bin width of 0.1 ms. We observe peaks of different intensities because the microchannel’s depth (30 µm) is larger than the focused laser waist. Molecules flowing through the channel at different depths traverse the spot at different vertical distances from the focused spot and are excited to different intensities.

We measured the extracted DNA molecules in the microchannels for different initial sample concentrations. For this, lambda DNA (48 kbp) was spiked into the buffer at three concentrations (30 ng/μL, 150 ng/μL, 300 ng/μL). The signals were collected over time, starting right after the liquid was dropped into the inlet and continuing for over 30 min as the DNA was being extracted. Figure 4c shows the DNA flow throughput in the microchannel for the three samples. In all time measurements, an initial rise is observed in the first 6 min. Afterwards, the values stabilize until they decline at the end of the 30 min.

In the initial phase, the pulled down beads get trapped in the incubation chamber and are heated up. The initial increase in DNA flow-through rate likely reflects the time required for DNA molecules to detach from the magnetic beads. Then, steadily, the concentration of extracted DNA increases until equilibrium is reached. After 20 to 25 min, the measured concentration of extracted DNA decreases because no new magnetic beads are introduced onto the chip, resulting in fewer magnetic beads being drawn into the lower stream and no additional DNA being extracted.

Figure 4d shows the total number of measured DNA peaks over 30 min for the three samples. There is a correlation between the initial DNA in the buffer and the peak throughput measured from the extracted DNA in the chip. For the sample with an initial DNA concentration of 30 ng/μL, 3694 peaks were measured in 30 min. For concentrations of 150 ng/μL and 300 ng/μL, 6210 and 7231 peaks were counted, respectively. These numbers were obtained by counting the fluorescent peaks recorded at the photon counter. These microchannel measurements provide a real-time, semi-quantitative monitor of relative DNA throughput that scales with initial concentration; absolute quantification is not yet achieved, as the counted peaks represent a flux that depends on both molecular concentration and local flow velocity. At higher concentrations, more than one molecule may simultaneously occupy the diffraction-limited laser spot, further complicating absolute counting. A full quantitative calibration would require either pressure-controlled flow with known standards, and/or measuring local flow velocity independently. This is identified as a key goal for future development of the platform.

### 3.5. Measurements in the Nanochannel Area for Fragment Length Analysis

**Figure 5:**
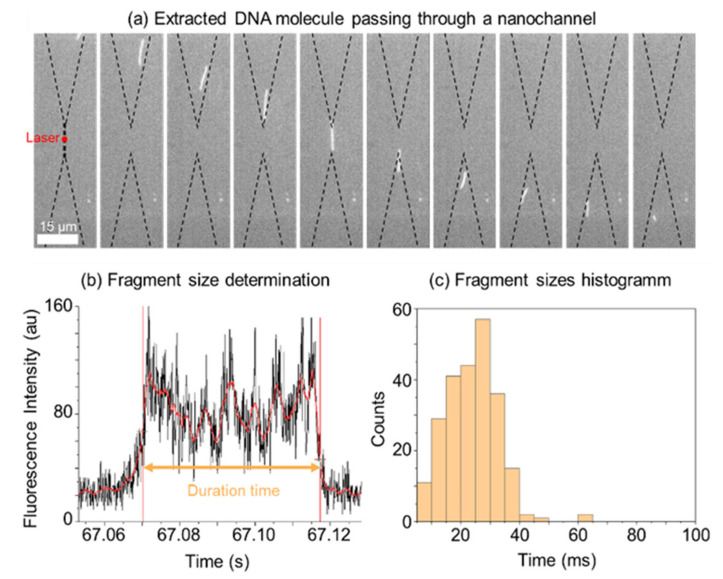
Characterization of extracted DNA at the single-molecule level in nanochannels. (**a**) Fluorescence images of a DNA molecule flowing through a nanochannel after extraction, where it can be seen pre-stretched in the inlet, and then flowing along the nanochannel. The laser is positioned at the center of a nanochannel and excites the molecules as the flow passes through. (**b**) Fluorescence fingerprint of a molecule of DNA passing through the laser, which is recorded in the photon counter as a step-like peak, and where the duration of the signal directly relates to the length of the DNA fragment. (**c**) Histogram showing the distribution of peak duration times for 238 DNA molecules extracted on-chip. The peak centered at 28.5 ms is consistent with intact full-length lambda DNA (48.5 kbp), as validated by the calibration in Figure A3c.

To analyze the extracted DNA at the single-molecule level, we employed nanochannels integrated into the chip. Within these nanochannels, DNA molecules are stretched, enabling direct measurement of features such as molecular length. Specifically, this on-chip method was used to assess potential DNA fragmentation resulting from the extraction process. Individual molecules were analyzed in real-time immediately after extraction using the previously demonstrated LADOM technique [31]. For this, a laser spot is focused at the center of a nanochannel. The DNA molecules flow from the microchannel into the 2D inlets, and then into the nanochannel, where they get stretched due to the reduced lateral dimensions. As they pass through the laser spot, their fluorescence is recorded in real time using a photon counter. The fluorescence signal from individual DNA molecules is recorded as step-like optical intensity peaks. Figure 5a shows a time-lapse sequence of a lambda DNA molecule entering and transiting a nanochannel, and Figure 5b presents a fluorescence time trace of a DNA molecule captured by the photon counter as it passes through the laser spot. Figure 5c shows a histogram of transit times for 238 lambda DNA molecules extracted from buffer.

**Figure A3:**
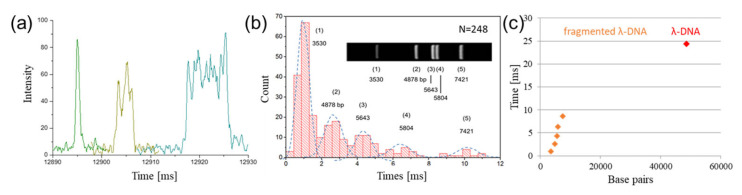
DNA length distribution measured using the LADOM system. Different DNA fragment lengths generate peaks with distinct temporal widths in the photodetector signal, measured in real time, as shown in the three selected time traces (**a**). A histogram of the temporal widths obtained from 248 measured molecules from a mixture of DNA fragments with six different lengths (**b**), from digested lambda DNA, where each Gaussian-shaped distribution corresponds to a specific fragment length. The average temporal duration for each one of the obtained fragment populations is plotted as a function of the expected fragment length in base pairs (**c**).

The transit time measured in the nanochannel is related to molecular length through the translocation velocity and the degree of stretching in the confinement. For the nanochannel dimensions used here (100 nm × 200 nm cross-section), lambda DNA (48.5 kbp, contour length ~16 µm unlabeled, ~20–22 µm when labelled), measured dynamically without relaxation [48], is expected to be linearized. The peak duration therefore scales with molecular length, though the relationship is not strictly linear for molecules longer than the nanochannel (10 µm), as the trailing end remains in the microchannel during transit [31]. Absolute length determination thus requires calibration with molecules of known length, as shown in Appendix A.3 for DNA of different lengths stretched in nanochannels (without the extraction part of the chip). The calibration curve in Figure A3c, obtained from five known fragment lengths spanning 3.5–7.4 kbp, provides the reference frame for interpreting transit times. The distribution in Figure 5c, centered at 28.5 ms, is consistent with the majority of the molecules retaining full length after extraction. The shorter-duration shoulder visible below ~15 ms may correspond to a minor population of folded full-length molecules, to a small fraction of fragmented DNA, or to molecules that were already fragmented in the input material.

### 3.6. On-Chip Extraction of DNA from Plasma

After demonstrating successful DNA extraction from buffer followed by subsequent on-chip DNA analysis, lambda DNA was spiked in human plasma and extracted using our developed microfluidic chip. In this case, the extraction efficiency was assessed by analyzing the recovered DNA off-chip, with standard methods.

**Figure 6:**
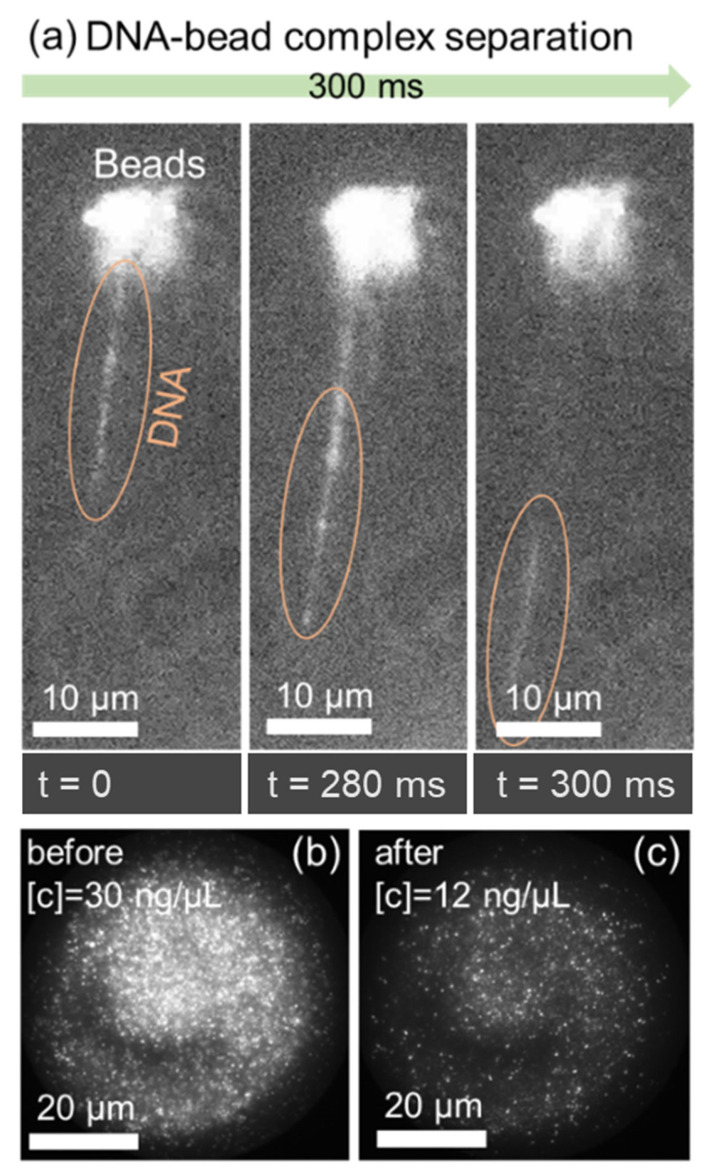
Separation of DNA–bead complex and resulting DNA extraction efficiency. (**a**) Fluorescence images showing the dissociation process of a DNA molecule (marked with a circle) from a cluster of magnetic beads (also marked in the first image) under the influence of heating in the elution buffer. The three images correspond to a time sequence, 300 ms long. The original video can be seen in the Supplementary Materials. (**b**) Fluorescence image of an initially impure sample of DNA in plasma with a concentration of 30 ng/µL. (**c**) Fluorescence image of the DNA extracted from this liquid sample using the extraction chip. The images in (**b**,**c**) were obtained using the same imaging conditions (exposure time, pixel binning, illumination intensity).

Figure 6 shows how a DNA fragment from a human plasma sample is released from the magnetic beads in the elution chamber of a chip (videos in the SI). The eluted DNA molecules continue to flow through the device and into the micro- and nanochannels, and can be recovered in one of the outlets.

For off-chip analysis, the extracted DNA was recovered from outlet D (see Figure 1) using filter paper inserted at the outlet. The DNA molecules can be recovered from the filter paper by gentle centrifugation. NanoDrop spectrofluorometer measurements were used to compare the DNA concentration before (30 ng/µL) and after recovery (12 ng/µL), indicating that approximately 60% of the DNA was lost during on-chip extraction and recovery of the material from the chip. This off-chip step is used here solely as an independent analytical validation; it is not an intrinsic part of the on-chip workflow. When the on-chip nanochannel readout is used as the analytical endpoint (as demonstrated in Section 3.5 for buffer), the filter paper step and its associated losses are eliminated entirely. The 40% overall extraction yield therefore reflects two discrete loss stages: ~14% of DNA–bead complexes remaining in the upper stream (Section 3.3) and ~46% lost during off-chip filter paper recovery. In addition, the extracted DNA was placed on a glass slide and observed under a fluorescence microscope. Figure 6b shows a fluorescence image of the DNA at its initial concentration (30 ng/µL) and recovered from the chip after extraction (12 ng/µL).

## 4. Discussion

This work demonstrates that DNA extraction and single-molecule analysis can be successfully integrated into an entirely passive lab-on-a-chip platform that operates without pumps, valves, or pressure-driven flow control, and integrated with on-chip single molecule analysis. The device relies solely on two external active components, a permanent magnet and a heating element, while maintaining straightforward handling and a rapid fabrication time of approximately 2 min per chip using direct UV imprinting. This combination of manufacturability, operational simplicity, and functional integration represents a significant step toward disposable and automated microfluidic analysis systems.

Under optimal conditions, an average bead capture efficiency of 85.6% was achieved. Real-time fluorescence measurements downstream of the elution chamber, provided detailed insight into the extraction kinetics. After sample introduction, the number of detected DNA molecules increased over the first minutes, then reached a steady state and eventually decreased as no further sample with beads entered the system, thus indicating a 30-min test time needed to extract and analyze the total inserted analysis liquid (10 µL). Total peak counts integrated over 30 min provide a semi-quantitative monitor of relative DNA throughput that scales with the initial concentration (30 ng/µL, 150 ng/µL, and 300 ng/µL), though absolute quantification requires further flow calibration (see Section 3.4). To assess DNA integrity after extraction, individual molecules were analyzed in nanochannels using the LADOM technique, which has been developed and validated by our group in prior work [31,32,34,35]. Hundreds of lambda DNA molecules were measured, yielding a transit-time distribution centered at 28.5 ms, consistent with most of the molecules retaining full length as validated by the calibration in Appendix A.3. A minor short-duration shoulder in the distribution may reflect folded molecules, a small fragmented fraction, or molecules that were already fragmented in the input material; the dominant population is full-length. The preservation of long DNA is critical for applications such as genome mapping and single-molecule structural characterization.

Several limitations of the current platform should be acknowledged. First, the 40% overall yield from plasma is comparable to or below typical commercial spin-column kits, which report 60–80% recovery from similar matrices but at the cost of larger sample volumes, multiple centrifugation steps, and longer processing times. The reduced yield reflects losses at two discrete stages: the 14% of DNA–bead complexes that remain in the upper stream and are discarded at outlet C, and the off-chip recovery step through filter paper and centrifugation, which accounts for approximately 46% loss as quantified by direct NanoDrop comparison (30 ng/µL input vs. 12 ng/µL recovered). We would like to emphasize that the off-chip filter paper step is not an intrinsic part of the device workflow—it was used here only as an independent analytical check. When the on-chip nanochannel readout is used as the analytical endpoint, this loss is eliminated entirely, and the effective yield is limited only by the 14% bead-transfer loss, bringing the potential on-chip yield to ~86%. Integrating a direct on-chip readout, as enabled by the nanochannel section, is therefore the main route toward closing the gap with conventional methods. Second, the microchannel-based real-time quantification is currently semi-quantitative: the focused laser probes only a sub-femtolitre volume at a single point in a channel of larger cross-section, so the counted peaks represent a flux that depends on both concentration and flow rate. We do observe a concentration-dependent scaling, but absolute concentration calibration will require either pressure-controlled flow or, preferably, a second laser spot to measure the local velocity directly. Third, nanochannel-based length measurement cannot yet match the absolute size accuracy of a calibrated gel ladder: our transit-time histograms resolve fragmented from intact molecules, but converting transit time to an absolute length requires a calibration curve recorded under identical stretching and flow conditions (Appendix A.3). What the nanochannel approach does offer over gel electrophoresis is single-molecule resolution without amplification and compatibility with sub-nanogram inputs, which is the regime where gel methods fail. The significance of single-molecule nanochannel analysis extends beyond what is achievable with conventional ensemble instruments. Gel electrophoresis and automated capillary electrophoresis systems (e.g., TapeStation) are excellent tools for quality control of extracted DNA when sufficient material is available. However, these methods require sample transfer out of the device, consume material that may be limited, and provide population-averaged size information rather than molecule-by-molecule measurements. Bulk quantification tools such as NanoDrop (spectrophotometric) and Qubit (fluorometric) provide accurate total DNA mass measurements but no length information; a sample containing short fragments and one containing intact long molecules give identical NanoDrop readings at the same mass concentration. The LADOM nanochannel technique, by contrast, measures each molecule individually as it flows through the device immediately after extraction, without amplification, without sample transfer, and on inputs well below the gel or spectrophotometric detection threshold. This single-molecule resolution could be relevant to cell-free DNA (cfDNA) fragmentomics in liquid biopsy [49,50], where the shape of the length distribution—not merely its mean—carries diagnostic information, and to forensic trace samples where material is insufficient for conventional analysis. The on-chip fluorescence readout is not proposed as a replacement for established quantification instruments such as NanoDrop or Qubit in well-equipped laboratory settings. Rather, its value lies in integration: concentration-dependent monitoring and single-molecule length analysis are performed within the same passive device as extraction, without removing the sample or transferring it to a benchtop instrument. This is directly relevant to point-of-care, portable, or field-deployed applications where NanoDrop and Qubit are unavailable, and to samples so scarce that any transfer step represents an unacceptable loss.

The current proof-of-concept demonstrates extraction from buffer and human plasma. The magnetophoretic separation principle is matrix-independent as the Reynolds numbers in these microchannels are very low (Re ≈ 0.0016 for the work presented here), and the commercial Dynabeads kit used is validated by the manufacturer for a range of biological matrices including whole blood, urine, and cell lysates. Non-biological contaminants that are not magnetically responsive would be removed by the laminar flow washing step along with the rest of the original sample matrix. The off-chip sample preparation is the same for both cell-free and cell-containing samples: (1) the sample, magnetic beads, and the kit’s combined lysis/binding buffer are mixed in a single tube and (2) loaded then into the chip. The lysis/binding buffer of the Dynabeads DNA DIRECT Universal Kit contains an ionic detergent (LiDS) that lyses cells in the same step in which DNA binds to the bead surface, so no separate pre-lysis incubation would be required for analyzing cultured cells, bacteria, or most clinical specimens. The off-chip workflow is therefore matrix-independent (mixing of kit with beads and incubation) and does not influence the on-chip protocol (pipetting, on-chip extraction and analysis of the DNA in the clean buffer). Experimental validation of the proposed device in additional matrices remains to be performed. A systematic device-to-device variability study under varying environmental conditions (humidity, ambient temperature) has not yet been performed; within individual experiments, laminar flow was verified before each run and was established in 100% of intact devices tested, and the bead-capture time series (Figure 3d) shows stable performance over 30 min, but standardization of inlet volume and ambient conditions will be important for future quantitative applications.

The lab-on-a-chip system offers fundamental advantages over the conventional two-step approach of vial-based extraction followed by transfer to a separate benchtop analytical instrument. By integrating sample processing and single-molecule analysis within a single passive device, it eliminates inter-step sample transfer, minimizes manual handling, reduces contamination risk, and removes the need for laboratory infrastructure beyond a fluorescence microscope. The 86% on-chip bead transfer efficiency is comparable to optimized vial-based magnetic bead protocols, while the 30 min passive run requires no operator intervention after sample loading—a meaningful advantage in point-of-care or field settings where skilled manual pipetting is unavailable. The system operates with minimal sample volumes (10 µL input), and the integrated single-molecule analytical capability enables characterization of scarce samples at concentrations and volumes where gel-based or spectrophotometric ensemble methods cannot reliably operate.

While the current performance is promising, future improvements in calibration of the molecular detection workflow are expected to enhance the device’s capabilities and extend the platform’s applicability to a range of analytical and diagnostic applications. To remove the dependence of absolute quantification on flow rate reproducibility, we are currently developing methods to monitor flow rate and molecular flow speeds, including a dual-laser-spot detection scheme. These will enable absolute concentration measurements independent of environmental flow rate variability. Determining the length of individual DNA molecules is essential not only because it provides direct information on molecular integrity, but also for several applications. For example, in liquid biopsy, accurate measurement of cell-free DNA fragmentation can serve as a biomarker for metastasis [49,50]. DNA fragmentation is also a predictive marker of tumor cell response to radiation therapy [51,52].

Future developments may include adding different chemicals (such as netropsin or actinomycin D) to the elution buffer for on-chip DNA barcoding and subsequent readout. Additionally, using a lysing buffer mixed with the beads (which is even commercially available) could further integrate an extra sample preparation step into the chip, enabling the analysis of more complex fluids following a similar protocol. Further integration with other on-chip functionalities, such as cell culture chambers, would allow a powerful platform for on-chip assays with real-time monitoring, for example, for infection or drug testing. We expect the devices to work also for other types of fluids, as the Reynolds number is still very low even for viscous liquids such as blood (between 0.02 and 2, depending on flow rates), and thus the operation principle, based on pulling down magnetic beads in the laminar flow chamber, should still work. Future work will aim to use the platform on real-world samples such as whole blood, urine, or environmental matrices. The primary target applications are point-of-care diagnostics—where minimal sample volumes, portability, and rapid turnaround are essential—and forensic or environmental analysis, where trace DNA from complex matrices must be extracted and characterized with minimal loss.

## 5. Conclusions

In summary, we have demonstrated a passive, polymer-based lab-on-a-chip integrating magnetophoretic DNA extraction with real-time single-molecule fluorescence analysis. The device achieves 86% on-chip bead transfer efficiency and 40% overall extraction yield from spiked plasma (from pipetted sample to recovered sample outside the chip), while preserving DNA integrity as confirmed by nanochannel fragment analysis. The two-minute fabrication process and passive operation make it well-suited for scalable, low-cost deployment. Future work will focus on improving extraction yield, quantitative flow calibration, and validation with clinically relevant samples.

## Figures and Tables

**Table 1:** Reactive-ion etching settings for the different structures patterned on the stamp, defined in three distinct steps.

Parameter	Microchannel	2D Inlets	Nanochannel
ICP (W)	300	400	400
SF_6_ (sccm)	30	50	50
C_4_F_8_ (sccm)	0	70	70
CHF_3_ (sccm)	72	0	0
O_2_ (sccm)	24	0	0
Temperature (°C)	30	0	0
Time (min)	30	10	1
Depth (μm)	30	0.2–0.8	0.1

## Data Availability

The original data sets presented in the study are available from the authors upon reasonable request.

## Supplementary Materials

The following supporting information can be downloaded at: https://www.mdpi.com/article/10.3390/nano16120732/s1. Two videos are available, where the DNA captured from plasma and released from beads in the elution chamber can be seen.

## Appendix A

### Appendix A.1. Thermal and Magnetic Field of the Microfluidic Device

The microfluidic device was analyzed using an inverted microscope, as shown in Figure A1. The microfluidic device itself is passive, while active components are applied externally. In this setup, the active components include a heating element and a permanent magnet. The heating element consists of a heating cartridge directed at the chip. The temperature distribution on the chip was monitored using a thermal camera, and the results are shown in Figure A1. When the heating element was set to 65 °C, a temperature of 62 °C was measured on the backside of the chip, with a heat distribution diameter of 5 mm. The incubation/elution chamber is fully contained within this 5 mm heated zone, confirming that the entire chamber reaches the target temperature. Additionally, the magnetic field was simulated to determine the optimal distance for placing the permanent magnet. The simulated magnetic field is depicted in Figure A1 (bottom right).

### Appendix A.2. Fabrication of Microfluidic Devices via Nanoimprint Lithography

Negative molds of the silicon structure are created in polymer-on-glass. These negative molds serve as master stamps for fabricating microfluidic devices. The microfluidic devices are produced using a nanoimprint lithography process that takes 2 min. A PMMA (polymethyl methacrylate) plate with pre-drilled holes at the inlets and outlets is used as the substrate. Figure A2 shows 3D microscope images of one such device. Figure A2a provides an overview of the entire device, including the purification and analysis regions. Figure A2b displays the laminar flow chamber, where magnetic beads are separated from the solution and transferred into a new buffer. Figure A2c shows the analysis area with the nanochannels, where the separated and fluorescently labeled DNA is detected.

### Appendix A.3. DNA Length Characterization Using LADOM

In previous experiments, we characterized the flow of DNA of different lengths in nanochannels. For this purpose, digested lambda DNA was mixed with TBE buffer and TOTO-3, and the DNA sample was introduced into a chip containing micro- and nanochannels (without the extraction part). DNA molecules of different lengths flowed through the channels and were detected using the LADOM system. The duration of the peaks obtained for the molecules correlates with the length of the DNA molecule, as shown in Figure A3a for individual signals obtained for different molecular fragments. The histogram in Figure A3b, obtained by measuring almost 250 molecules, shows Gaussian-shaped distributions for different fragment lengths (3530 bp, 4878 bp, 5643 bp, 5804 bp, 7421 bp). The graph in Figure A3c shows the relation between the fragment length (base pairs) and the measured peak width (ms) for the fragments and for intact lambda DNA. The relation is not linear, as longer molecules interact more strongly with the inlets and outlets. This calibration curve helps to interpret the transit-time histogram of Figure 5c (Section 3.5): the peak centered at 28.5 ms is consistent with full-length 48.5 kbp lambda DNA according to this curve. The calibration further demonstrates that fragmented molecules as short as ~3.5 kbp are clearly resolved from full-length molecules, confirming the sensitivity of the method.

In the work presented in the main text, we spiked intact lambda DNA in the liquids; thus, after statistical analysis, we expected to obtain a single peak in the distribution for non-fragmented DNA, or multiple peaks in the histogram if the DNA would have been damaged during the process. The histogram shown in Figure 5c shows that the majority of the measured peaks had a duration of around 20 to 35 ms, as expected for intact molecules.

## References

[1] Hawkins T.L., O’Connor-Morin T., Roy A., Santillan C. (1994). DNA purification and isolation using a solid-phase. Nucleic Acids Res..

[2] Tan S.C., Yiap B.C. (2009). DNA, RNA, and protein extraction: The past and the present. J. Biomed. Biotechnol..

[3] Wilson K. (2001). Preparation of genomic DNA from bacteria. Curr. Protoc. Mol. Biol..

[4] Dairawan M., Shetty P.J. (2020). The Evolution of DNA Extraction Methods. Am. J. Biomed. Sci. Res..

[5] Israel D.I. (1993). A PCR-based method for high stringency screening of DNA libraries. Nucleic Acids Res..

[6] Meyer M., Briggs A.W., Maricic T., Hober B., Hoffner B., Krause J., Weihmann A., Paabo S., Hofreiter M. (2008). From micrograms to picograms: Quantitative PCR reduces the material demands of high-throughput sequencing. Nucleic Acids Res..

[7] Kuchler E.C., Tannure P.N., Falagan-Lotsch P., Lopes T.S., Granjeiro J.M., Amorim L.M. (2012). Buccal cells DNA extraction to obtain high quality human genomic DNA suitable for polymorphism genotyping by PCR-RFLP and Real-Time PCR. J. Appl. Oral Sci..

[8] Andréasson H., Gyllensten U., Allen M. (2002). Real-Time DNA Quantification of Nuclear and Mitochondrial DNA in Forensic Analysis. BioTechniques.

[9] Elphinstone M.S., Hinten G.N., Anderson M.J., Nock C.J. (2003). An inexpensive and high-throughput procedure to extract and purify total genomic DNA for population studies. Mol. Ecol. Notes.

[10] Lu Y., Bianco P. (2021). High-yield purification of exceptional-quality, single-molecule DNA substrates. J. Biol. Methods.

[11] Smith K., Diggle M.A., Clarke S.C. (2003). Comparison of commercial DNA extraction kits for extraction of bacterial genomic DNA from whole-blood samples. J. Clin. Microbiol..

[12] Maukonen J., Simoes C., Saarela M. (2012). The currently used commercial DNA-extraction methods give different results of clostridial and actinobacterial populations derived from human fecal samples. FEMS Microbiol. Ecol..

[13] Stuppia L., Antonucci I., Palka G., Gatta V. (2012). Use of the MLPA assay in the molecular diagnosis of gene copy number alterations in human genetic diseases. Int. J. Mol. Sci..

[14] Simon-Sanchez J., Scholz S., Fung H.C., Matarin M., Hernandez D., Gibbs J.R., Britton A., de Vrieze F.W., Peckham E., Gwinn-Hardy K. (2007). Genome-wide SNP assay reveals structural genomic variation, extended homozygosity and cell-line induced alterations in normal individuals. Hum. Mol. Genet..

[15] Poole C.F. (2003). New trends in solid-phase extraction. TrAC Trends Anal. Chem..

[16] Żwir-Ferenc A., Biziuk M. (2006). Solid Phase Extraction Technique–Trends, Opportunities and Applications. Pol. J. Environ. Stud..

[17] Oblath E.A., Henley W.H., Alarie J.P., Ramsey J.M. (2013). A microfluidic chip integrating DNA extraction and real-time PCR for the detection of bacteria in saliva. Lab Chip.

[18] Zhang Y., Park S., Yang S., Wang T.H. (2010). An all-in-one microfluidic device for parallel DNA extraction and gene analysis. Biomed. Microdevices.

[19] Cho Y.K., Lee J.G., Park J.M., Lee B.S., Lee Y., Ko C. (2007). One-step pathogen specific DNA extraction from whole blood on a centrifugal microfluidic device. Lab Chip.

[20] Shaw K.J., Joyce D.A., Docker P.T., Dyer C.E., Greenway G.M., Greenman J., Haswell S.J. (2011). Development of a real-world direct interface for integrated DNA extraction and amplification in a microfluidic device. Lab Chip.

[21] Fernandez-Cuesta I., Llobera A., Ramos-Payan M. (2022). Optofluidic systems enabling detection in real samples: A review. Anal. Chim. Acta.

[22] Zhu C., Hu A., Cui J., Yang K., Zhu X., Liu Y., Deng G., Zhu L. (2019). A Lab-on-a-Chip Device Integrated DNA Extraction and Solid Phase PCR Array for the Genotyping of High-Risk HPV in Clinical Samples. Micromachines.

[23] Chung Y.C., Jan M.S., Lin Y.C., Lin J.H., Cheng W.C., Fan C.Y. (2004). Microfluidic chip for high efficiency DNA extraction. Lab Chip.

[24] Campos C.D.M., Gamage S.S.T., Jackson J.M., Witek M.A., Park D.S., Murphy M.C., Godwin A.K., Soper S.A. (2018). Microfluidic-based solid phase extraction of cell free DNA. Lab Chip.

[25] Nestorova G.G., Hasenstein K., Nguyen N., DeCoster M.A., Crews N.D. (2017). Lab-on-a-chip mRNA purification and reverse transcription via a solid-phase gene extraction technique. Lab Chip.

[26] Koo K.M., Wee E.J.H., Wang Y., Trau M. (2017). Enabling miniaturised personalised diagnostics: From lab-on-a-chip to lab-in-a-drop. Lab Chip.

[27] Schiebelhut L.M., Abboud S.S., Gomez Daglio L.E., Swift H.F., Dawson M.N. (2017). A comparison of DNA extraction methods for high-throughput DNA analyses. Mol. Ecol. Resour..

[28] Duarte G.R., Price C.W., Augustine B.H., Carrilho E., Landers J.P. (2011). Dynamic solid phase DNA extraction and PCR amplification in polyester-toner based microchip. Anal. Chem..

[29] Xu Y., Vaidya B., Patel A.B., Ford S.M., McCarley R.L., Soper S.A. (2003). Solid-phase reversible immobilization in microfluidic chips for the purification of dye-labeled DNA sequencing fragments. Anal. Chem..

[30] Fu Y., Zhou X., Xing D. (2017). Lab-on-capillary: A rapid, simple and quantitative genetic analysis platform integrating nucleic acid extraction, amplification and detection. Lab Chip.

[31] Esmek F.M., Erichlandwehr T., Mors D.H.B., Czech-Sioli M., Therre M., Günther T., Grundhoff A., Fischer N., Fernandez-Cuesta I. (2021). Real time, in-line optical mapping of single molecules of DNA. Biosens. Bioelectron. X.

[32] Esmek F.M., Bayat P., Perez-Willard F., Volkenandt T., Blick R.H., Fernandez-Cuesta I. (2019). Sculpturing wafer-scale nanofluidic devices for DNA single molecule analysis. Nanoscale.

[33] Esmek F.M., Erichlandwehr T., Brkovic N., Pranzner N.P., Teuber J.P., Fernandez-Cuesta I. (2022). Pillar-structured 3D inlets fabricated by dose-modulated e-beam lithography and nanoimprinting for DNA analysis in passive, clogging-free, nanofluidic devices. Nanotechnology.

[34] Esmek F.M., Grzybeck P., Nasri R., Tiwari S., Fernandez-Cuesta I. (2024). Flow Behavior Characterization of DNA Molecules in Passive Nanofluidic Devices. IEEJ Trans. Electr. Electron. Eng..

[35] Czech-Sioli M., Günther T., Therre M., Spohn M., Indenbirken D., Theiss J., Riethdorf S., Qi M., Alawi M., Wülbeck C. (2020). High-resolution analysis of Merkel Cell Polyomavirus in Merkel Cell Carcinoma reveals distinct integration patterns and suggests NHEJ and MMBIR as underlying mechanisms. PLoS Pathog..

[36] Fernandez-Cuesta I., Laura Palmarelli A., Liang X., Zhang J., Dhuey S., Olynick D., Cabrini S. (2011). Fabrication of fluidic devices with 30 nm nanochannels by direct imprinting. J. Vac. Sci. Technol. B.

[37] Gunther K., Mertig M., Seidel R. (2010). Mechanical and structural properties of YOYO-1 complexed DNA. Nucleic Acids Res..

[38] Kim Y., Kim K.S., Kounovsky K.L., Chang R., Jung G.Y., dePablo J.J., Jo K., Schwartz D.C. (2011). Nanochannel confinement: DNA stretch approaching full contour length. Lab Chip.

[39] Thermo Fisher Scientific (2026). Dynabeads DNA DIRECT Universal Kit. Product No. 63006.

[40] Zhou J., Wang Y., Menard L.D., Panyukov S., Rubinstein M., Ramsey J.M. (2017). Enhanced nanochannel translocation and localization of genomic DNA molecules using three-dimensional nanofunnels. Nat. Commun..

[41] Marie R., Pedersen J.N., Bauer D.L., Rasmussen K.H., Yusuf M., Volpi E., Flyvbjerg H., Kristensen A., Mir K.U. (2013). Integrated view of genome structure and sequence of a single DNA molecule in a nanofluidic device. Proc. Natl. Acad. Sci. USA.

[42] Marie R., Pedersen J.N., Baerlocher L., Koprowska K., Podenphant M., Sabatel C., Zalkovskij M., Mironov A., Bilenberg B., Ashley N. (2018). Single-molecule DNA-mapping and whole-genome sequencing of individual cells. Proc. Natl. Acad. Sci. USA.

[43] Lima D.C., Nyberg L.K., Westerlund F., Batistuzzo de Medeiros S.R. (2018). Identification and DNA annotation of a plasmid isolated from Chromobacterium violaceum. Sci. Rep..

[44] Ostergaard P.F., Matteucci M., Reisner W., Taboryski R. (2013). DNA barcoding via counterstaining with AT/GC sensitive ligands in injection-molded all-polymer nanochannel devices. Analyst.

[45] Nyberg L.K., Persson F., Berg J., Bergstrom J., Fransson E., Olsson L., Persson M., Stalnacke A., Wigenius J., Tegenfeldt J.O. (2012). A single-step competitive binding assay for mapping of single DNA molecules. Biochem. Biophys. Res. Commun..

[46] Lam E.T., Hastie A., Lin C., Ehrlich D., Das S.K., Austin M.D., Deshpande P., Cao H., Nagarajan N., Xiao M. (2012). Genome mapping on nanochannel arrays for structural variation analysis and sequence assembly. Nat. Biotechnol..

[47] McCaffrey J., Sibert J., Zhang B., Zhang Y., Hu W., Riethman H., Xiao M. (2016). CRISPR-CAS9 D10A nickase target-specific fluorescent labeling of double strand DNA for whole genome mapping and structural variation analysis. Nucleic Acids Res..

[48] He Q.H., Ranchon H., Carrivain P., Viero Y., Lacroix J., Blatché C., Daran E., Victor J.M., Bancaud A. (2013). Conformational Manipulation of DNA in Nanochannels Using Hydrodynamics. Macromolecules.

[49] Ding S.C., Lo Y.M.D. (2022). Cell-Free DNA Fragmentomics in Liquid Biopsy. Diagnostics.

[50] Joosse S.A., Pantel K. (2022). Circulating DNA and Liquid Biopsies in the Management of Patients with Cancer. Cancer Res..

[51] Huang R.X., Zhou P.K. (2020). DNA damage response signaling pathways and targets for radiotherapy sensitization in cancer. Signal Transduct. Target. Ther..

[52] Penninckx S., Pariset E., Cekanaviciute E., Costes S.V. (2021). Quantification of radiation-induced DNA double strand break repair foci to evaluate and predict biological responses to ionizing radiation. NAR Cancer.

